# Personality vulnerabilities and adverse event reporting in phase 1 clinical studies

**DOI:** 10.1186/s13063-024-08321-4

**Published:** 2024-07-18

**Authors:** Joanna Skommer, Krish Gunesh, Thomas M. Polasek

**Affiliations:** 1 CMAX Clinical Research, 21 North Terrace, Adelaide, SA 5000 Australia; 2 Fusion Clinical Research, 64a Fullarton Road, Norwood, SA 5067 Australia; 3 Central Adelaide Health Network, SA Health, Adelaide, Australia; 4 Centre for Medicine Use and Safety, Monash University, Melbourne, Australia

**Keywords:** Phase 1 clinical trials, Healthy volunteers, Personality

## Abstract

**Background:**

Phase 1 clinical trials involve rigorous safety monitoring to identify any adverse effects of investigational treatments. There is growing evidence that healthy volunteers recruited in these studies may differ with respect to personality traits from the general population. This, in turn, may have a significant impact on the reporting of adverse events, particularly in trials investigating psychoactive treatments, including the psychedelic substances.

**Main body:**

This analysis stems from our combined experience as investigators in phase 1 clinical trials and conveys an experiential understanding of the impact of psychological heterogeneity on study participation, reporting of adverse events and study outcomes.

**Conclusion:**

Participant variability due to psychological characteristics is regularly overlooked in phase 1 clinical trials and may significantly impact on reporting of the adverse events. In our opinion, healthy volunteers who present for these studies should not only be defined by the absence of past or current medical and psychiatric illness but also characterised by their psychological attributes.

## Background

There are rising concerns in the literature that harm from interventions is insufficiently documented in clinical trials [1]. In response to the growing interest in psychedelic drugs for the treatment of psychiatric disorders and the booming clinical research in the field, this commentary is a cautionary note to doctors who conduct phase 1 clinical studies about how psychological heterogeneity may impact study participation and results, particularly reporting of adverse events.

Phase 1 clinical studies are fundamental for the development of new drugs [2]. A defining feature of these studies is the participation of healthy volunteers (HVs) who, in principle, lack characteristics that disrupt on-study activities and negatively influence study results. This ensures that the pharmacokinetics and safety (part of pharmacodynamics) of new drugs are determined accurately without interference from concomitant pathological conditions [3]. Participants must be in excellent health and able to adhere to study protocols, abstaining from prohibited substances (e.g. prescribed, over-the-counter and recreational drugs, alcohol, and smoking) and complying with all assessments and procedures. Prior to enrolment, comprehensive screening of potential participants is conducted by applying protocol inclusion and exclusion criteria. However, emphasis during screening is placed on physical health rather than psychological characteristics. Currently, based on our experience, no specific procedures are being routinely implemented in phase 1 clinical studies to ensure that HVs are representative of the psychological wellbeing and traits of the general population.

## The human factor: personalities of healthy volunteers

Recruiting HVs from large non-clinical populations seemingly conforms to the goal of random sampling. However, the willingness to participate in clinical research, as well as the propensity to respond to advertisements and be attracted by incentives, such as financial renumeration, may distinguish HVs from the general population. While economic gain is one of the strongest motivators, other factors that influence willingness to volunteer in clinical trials include curiosity, wish to meet people, and choice for risky activities. The earliest suggestion that volunteers differ in personality features from the general population was presented in the 1950s [4]. In 1975, Rosenthal and Rosenow also suggested that clinical research volunteers tend to be more unconventional and more arousal- and approval-seeking than non-volunteers [5]. Since then, multiple further studies, including those employing standardised personality rating scales (e.g. the Eysenck Personality Questionnaire and Freiburg Personality Inventory), have demonstrated significant differences in personality functioning between participants in phase 1 clinical studies and the general population [6–13]. Overall, lower levels of neuroticism and higher levels of extraversion, psychoticism, and openness to experience, as well as disinhibition, were repeatedly noted.

Such characteristics may also vary depending on the nature of the research and the types of tasks involved. Gustavsson and co-workers observed that individuals who chose to participate in a study with a potentially painful procedure, a lumbar puncture, tended to be more impulsive than those who declined [14]. Likewise, the research topic advertised during recruitment may differentially attract interest, thus biasing respondents in a way that conforms to personality dimensions [15]. To this point, we hypothesise that the psychological characteristics of HVs enrolling in phase 1 clinical studies of psychedelic drugs, such as psilocybin, 3,4-methylenedioxymethamphetamine (MDMA), tetrahydrocannabinol (THC), lysergic acid diethylamide (LSD), ibogaine and iprocin, may differ significantly from those participating in other phase 1 clinical studies. This is pertinent given the marked increase in psychedelic drug research globally following regulatory approval of psilocybin-assisted psychotherapy for treatment-resistant depression in early 2023 [16].

## Personality traits affect adverse effect reporting

Personality traits are a poorly studied factor that may be relevant to the reporting of adverse effects. An understanding of the moderating role of individual personality traits on the frequency and intensity of adverse effect reporting is still at its infancy, and the scale of the problem has not been systematically and consistently addressed in drug trial research. Webster and co-authors [17] assessed 89 studies to identify factors contributing to adverse effects resulting from a psychologically mediated nocebo response. Their study identified unrealistic dose expectations of participants as predictors of nocebo response and suggested that these should be reduced especially ‘for persons with at-risk personality types’, acknowledging that the specifics of those personality types require further study.

There is compelling evidence that personality traits shape people’s subjective interpretations of their health status [18]. People with high scores on neuroticism scales are more likely to report medically unfounded somatic complaints, have catastrophic thoughts about their symptoms, and ask for medical help [19–21]. In addition, higher conscientiousness has been found to be related to a bias toward reporting disease among persons who do not meet the clinical criteria for disease.

In line with research on the association between personality traits and somatisation, higher levels of conscientiousness and neuroticism are also linked with reporting of adverse events to medications in population-based studies [22]. On the other hand, people with higher level of agreeableness are more likely to report pain relief from a placebo. So far, only a few studies conducted on small samples have specifically examined the relationships between personality traits and the reporting or developing of adverse effects in clinical trials. It has been suggested that less neurotic participants report fewer adverse effects, while subjects with higher negative affectivity report increased drug-related symptoms, known as the negative Hawthorne effect [23–26]. Nevertheless, based on the currently available evidence, it is challenging to definitively determine the exact connection between the level of neuroticism and the accuracy of AE reporting. It is plausible that lower level of neuroticism leads to AE under-reporting. Alternatively, we can postulate that participants with higher level of neuroticism over-report AEs, while their comparator group (people with lower level of neuroticism) report them accurately.

**Fig. 1:**
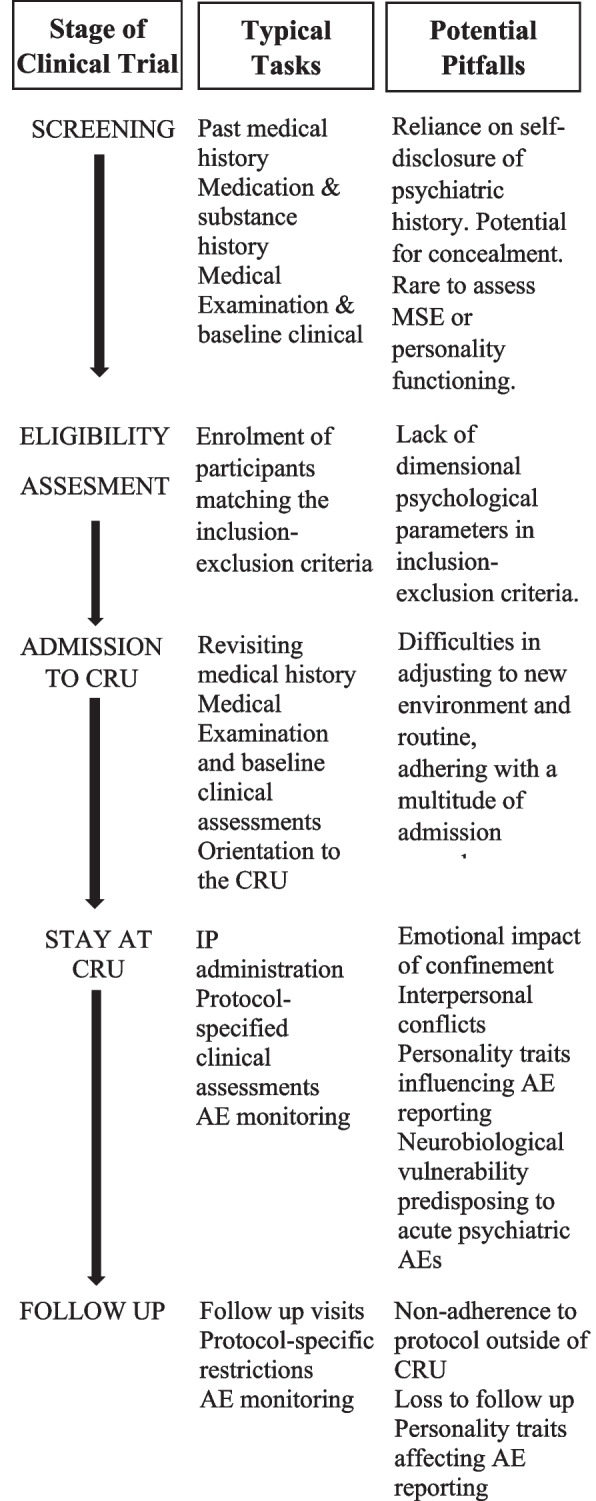
General design of phase 1 clinical trials and potential for impact of personality vulnerabilities. AE, adverse event; CRU, clinical research unit; IP, investigational product; MSE, mental state examination

The potential difficulties arising from personality vulnerabilities during phase 1 clinical studies are summarised in Fig. 1. Apart from vulnerability to interpersonal conflicts, which in our combined experience as phase 1 clinical trial investigators can predispose participants to early withdrawal and acute worsening of mental well-being, personality traits are well known to influence pharmacodynamics, particularly for drugs with central nervous system effects [27]. This is particularly important in phase 1 clinical studies because adverse effect reporting gives an initial indication of drug safety, a primary endpoint in most early phase studies [23–26].

Interestingly, there is some evidence that genetic polymorphisms of the enzyme CYP2D6 are associated with different personality types, putatively by variable metabolism of serotonin and dopamine precursors, thus indirectly linking the pharmacokinetics of drugs eliminated by this enzyme to personality [28]. Different gastric emptying rates between personality types have also been considered as a source of pharmacokinetic variability [29].

## Conclusions and recommendations

To summarise, the criteria for determining the ‘normality’ of HVs in phase 1 clinical studies are often subjective despite protocol inclusion and exclusion criteria. Participant variability due to psychological characteristics is regularly overlooked and may significantly affect the reporting of adverse events. In our opinion, more research is warranted to examine the psychological profiles of HVs who present for these studies, in connection with AE reporting.

Personality should be dimensionally assessed by applying the Five Factor Model of general personality structure or its instantiation, the DSM-5 Alternative Model of Personality Disorders (AMPDs) [30]. This would allow incorporating quotas of different personality traits to be included in the cohort design, in tandem guiding the development of participant-focused communication strategies and recruitment approaches to increase participation of personality types which may currently be underrepresented in clinical trials. It would also allow post-hoc analysis of personality types against pharmacokinetic and pharmacodynamic data (e.g. adverse effects). Past psychiatric history should be determined at screening to ensure validity of study results and to prevent the emotional burden of research participation for already vulnerable individuals. The reliance on self-disclosure or cross-sectional mental state examination may be insufficient to guard against participants masking their history. Hence, we suggest utilisation of approaches such as obtaining health summary information for prospective participants from their primary health providers, obtaining consent to review clinical records at baseline, and development of databases of phase I clinical trial participants with longitudinal monitoring of behaviours of concern. Our recommendations are warranted for all phase 1 clinical studies but are particularly important for early clinical research with psychedelic drugs, including phase 1 as well as phase 2 trials [31], where adverse effect reporting and determination of relatedness to investigational drugs may be further complicated by the concomitant administration of psychological therapy.

## Data Availability

Not applicable.

## Abbreviations

AE: Adverse event
AMPDs: Alternative Model of Personality Disorders
DSM 5: Diagnostic and Statistical Manual 5
HV: Healthy volunteers
LSD: Lysergic acid diethylamide
MDMA: 3,4-Methylenedioxymethamphetamine
THC: Tetrahydrocannabinol

## References

[1] Abdel SC, Maher CG, Furmage AM (2022). Strengthening the reporting of harms of all interventions in clinical trials. Med J Aust.

[2] Polasek TM, Schuck V (2023). Improving the efficiency of clinical pharmacology studies. Clin Pharmacol Drug Dev.

[3] Doogue MP, Polasek TM (2013). The ABCD of clinical pharmacokinetics. Ther Adv Drug Saf.

[4] Pollin W, Perlin S (1958). Psychiatric evaluation of "normal control" volunteers. Am J Psychiatry.

[5] Rosenthal R, Rosnow RL (1965). The volunteer subject. Hum Relat.

[6] Walsh JA, Nash MM (1978). Personality characteristics of volunteers for medical research. Crim Justic Behav.

[7] Cowles M, Davis C (1987). The subject matter of psychology: Volunteers. Br J Soc Psychol.

[8] Cami J, Llorente M, Farre M (1989). Personality of healthy volunteers participating in phase I clinical trials. Pers Individ Differ.

[9] Ball CJ, McLaren PM, Morrison PJ (1993). The personality structure of ‘normal’ volunteers. Br J Clin Pharmacol.

[10] Berto D, Milleri S, Squassante L (1996). Evaluation of personality as a component of the healthy condition of volunteers participating in phase I studies. Eur J Clin Pharmacol.

[11] Tishler C, Apseloff G, Bartholomae S (2007). Are normal healthy research volunteers psychologically healthy? A pilot investigation. Exp Clin Psychopharmacol.

[12] Wei Y, Li H, Wang H, Zhang S (2018). Psychological status of volunteers in a phase I clinical trial assessed by symptom checklist 90 (SCL-90) and Eysenck Personality Questionnaire (EPQ). Med Sci Monit.

[13] Farre M, Lamas X, Cami J (1995). Sensation seeking amongst healthy volunteers participating in phase I clinical trials. Br J Clin Pharmacol.

[14] Gustavsson JP, Asberg M (2007). The healthy control subject in psychiatric research: impulsiveness and volunteer bias. Acta Psychiatr Scand.

[15] Pieters MS, Jennekens-Schinkel A, Shoemaker HC (1992). Self-selection for personality variables among healthy volunteers. Br J Clin Pharmacol.

[16] Royal Australian and New Zealand College of psychiatrists. Clinical Memorandum Therapeutic use of MDMA for PTSD and psilocybin for treatment resistant depression. 2023. https://www.ranzcp.org/getmedia/0cf57ea2-0bd7-4883-9155-d2ba1958df86/cm-therapeutic-use-of-mdma-for-ptsd-and-psilocybin-for-treatment-resistant-depression.pdf. Accessed Dec 2023.

[17] Webster RK, Weinman J, Rubin GJ (2016). A systematic review of factors that contribute to nocebo effects. Health Psychol.

[18] Kööts-Ausmees L, Schmidt M, Esko T (2016). The role of the five factor personality traits in self-reported general health. Eur J Personal.

[19] Watson D (1989). Strangers’ ratings of five robust personality factors: Evidence of a surprising convergence with self-report. J Person Soc Psychology.

[20] Costa PT, McCrae RR (1987). Neuroticism, somatic complaints, and disease: is the bark worse than the bite?. J Pers.

[21] Jerram KL, Coleman PG (1999). The big five personality traits and reporting of health problems and health behaviour in old age. Br J Health Psychol.

[22] Realo A, Van Middendorp H, Koots-Ausmees L (2018). Role of personality traits in reporting the development of adverse drug reactions: a prospective cohort study of the Estonian general population. BMJ Open.

[23] Almeida L, Falcão A, Vaz-da-Silva M (2008). Personality characteristics of volunteers in Phase 1 studies and likelihood of reporting adverse events. Int J Clin Pharmacol Ther.

[24] Almeida L, Kasdan TB, Nunes T (2008). Who volunteers for phase I clinical trials? Influences of anxiety, social anxiety and depressive symptoms on self-selection and the reporting of adverse events. Eur J Clin Pharmacol.

[25] Foster JM, Sanderman R, van der Molen T (2008). Personality influences the reporting of side effects of inhaled corticosteroids in asthma patients. J Asthma.

[26] Davis C, Ralevski E, Kennedy S (1995). The role of personality factors in the reporting of side effect complaints to moclobemide and placebo: a study of healthy male and female volunteers. J Clin Psychopharmacol.

[27] Amare AT, Schubert KO, Tekola-Ayele F (2018). Association of polygenic score for personality traits and response to selective serotonin re-uptake inhibitors in patients with major depressive disorder. Front Psychiatry.

[28] Xu F (2007). Effect of personality type on pharmacodynamics through changing pharmacokinetics. Med Hypotheses.

[29] Tishler C, Bartholomae S, Rhodes A (2005). Personality profiles of normal healthy research volunteers: a potential concern for clinical drug trial investigators?. Med Hypotheses.

[30] Thomas A (2020). The Alternative Model of Personality Disorders (AMPD) from the Perspective of the Five-Factor Model. Psychopathology.

[31] Rucker J, Jafari H, Mantingh T (2021). Psilocybin-assisted therapy for the treatment of resistant major depressive disorder (PsiDeR): protocol for a randomised, placebo-controlled feasibility trial. BMJ Open.

